# Osteochondral flake refixation using the parachute technique yields favourable clinical and radiological outcomes following acute patellar dislocation: A case series at short‐ to mid‐term follow‐up

**DOI:** 10.1002/jeo2.70894

**Published:** 2026-09-09

**Authors:** Romed Peter Vieider, Yannick Ehmann, Svenja Höger, Lorenz Fritsch, Charlotte Schweitzer, Armin Runer, Alexander Wolfgang Marka, Sebastian Siebenlist, Julian Mehl

**Affiliations:** ^1^ Department of Sports Orthopaedics TUM University Hospital, Klinikum rechts der Isar, Technical University of Munich Munich Germany; ^2^ Institute for Diagnostic and Interventional Radiology, School of Medicine and Health, TUM Klinikum Technical University of Munich (TUM) Munich Germany; ^3^ ECOM Munich Germany

**Keywords:** cartilage, flake fracture, MOCART, parachute technique, patellar dislocation, patellofemoral instability, refixation

## Abstract

**Purpose:**

To evaluate short‐ to mid‐term clinical and radiological outcomes following osteochondral flake refixation with transpatellar, absorbable sutures (parachute technique) combined with concomitant medial patellofemoral ligament (MPFL) reconstruction after acute patellar dislocation. It was hypothesized that the parachute technique would yield favourable clinical and functional outcomes and demonstrate good osteochondral integration on radiographic follow‐up.

**Methods:**

A retrospective case series was conducted, including patients who underwent osteochondral flake refixation using the parachute technique following acute patellar dislocation between January 2012 and November 2023 at a minimum follow‐up of 24 months. Clinical outcomes were assessed using the Visual Analogue Scale (VAS), Tegner Activity Scale (TAS), Kujala Score, International Knee Documentation Committee (IKDC) subjective score and Knee Injury and Osteoarthritis Outcome Score (KOOS). Radiological outcomes were evaluated using the Magnetic Resonance Observation of Cartilage Repair Tissue (MOCART) 2.0 knee score on 3.0‐T magnetic resonance imaging (MRI) by three independent raters and intraclass correlation coefficient (ICC) was assessed.

**Results:**

Of 26 identified patients, 20 (77%) were available at final follow‐up (mean age 20.8 ± 5.3 years; mean follow‐up 62.2 ± 20.5 months; mean defect size 2.8 ± 1.5 cm^2^). Clinical outcome scores resulted in a mean VAS of 0.5 ± 1.6, TAS of 6 (interquartile range 4–7), Kujala Score of 89.4 ± 12.5, IKDC of 86.7 ± 14.3 and total KOOS of 87.8 ± 14.1. Of all patients, 95% were satisfied with the overall result, and 90% would be willing to undergo the procedure again if necessary. Two patients (10%) required revision surgery (one patella re‐dislocation, one failed osteochondral integration). The mean MOCART 2.0 score was 84 ± 8 (range: 75–95), indicating good to excellent osteochondral integration. Inter‐rater reliability across the three raters was almost perfect (ICC = 0.84; 95% confidence interval 0.79–0.96).

**Conclusion:**

The parachute technique yields favourable clinical and functional outcomes at short‐ to mid‐term follow‐up and demonstrates good to excellent osteochondral integration on MRI following acute patellar dislocation. The technique represents a reliable, simple and single‐stage option for the treatment of retropatellar osteochondral flake fractures.

**Level of Evidence:**

Level IV, case series.

## Graphical abstract

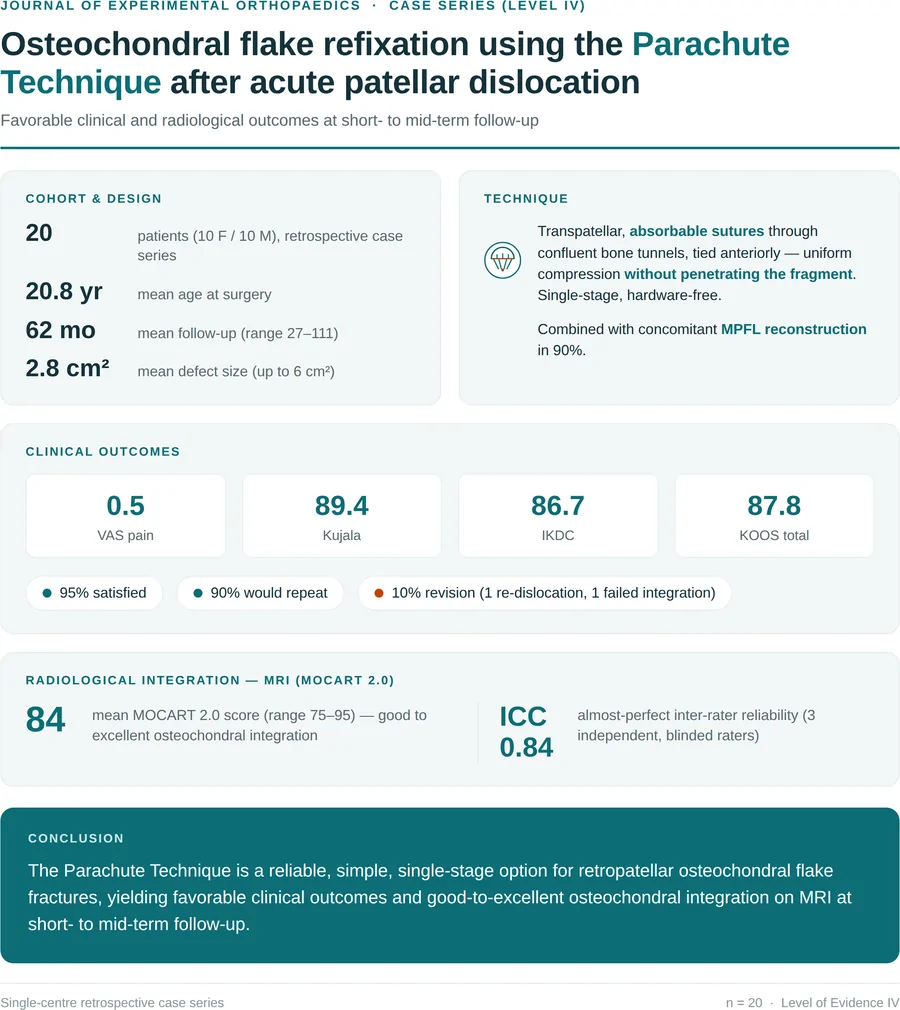

**Abbreviations**
ADL: Activities of Daily Living
BMI: body mass index
CI: confidence interval
cm^2^
: square centimetre
ICC: intraclass correlation coefficient
IKDC: International Knee Documentation Committee
IRB: institutional review board
kg: kilograms
KOOS: Knee Injury and Osteoarthritis Outcome Score
MOCART: Magnetic Resonance Observation of Cartilage Repair Tissue
MPFL: medial patellofemoral ligament
MRI: magnetic resonance imaging
PROM: patient‐reported outcome measure
ROM: range of motion
SD: standard deviation
TAS: Tegner Activity Scale
VAS: Visual Analogue Scale

## INTRODUCTION

Patellar dislocation is a common injury in orthopaedic practice, predominantly affecting young and physically active individuals [4, 17, 26, 29]. Chondral injuries are reported in up to 95% of cases and are defined as osteochondral fractures of the retropatellar surface (flake fractures), occurring in up to 58% of patients [4, 16, 23, 29]. Registry data have further characterized the incidence and current treatment strategies for patellar instability [11, 12]. Chondral or osteochondral lesions should be repaired when the defect in the patellofemoral joint contact area measures at least 1 cm^2^, as there is a risk of early patellofemoral osteoarthritis [2, 3]. In cases of large osteochondral defects and intact fragments (>2 cm^2^) where the surrounding cartilage allows for adequate embedding, primary open re‐fixation is one preferred approach [7, 19, 31]. This method preserves native hyaline cartilage in a single‐stage procedure [4, 7]. Various fixation techniques have been described, including bioabsorbable screws, pins and suture‐based constructs, although no consensus exists on the superiority of a specific technique [8, 14, 17].

The parachute technique is a simple and cost‐effective single‐stage method for the refixation of retropatellar flake fractures [19]. It uses transpatellar, absorbable sutures that are passed through confluent bone tunnels and tied anteriorly, thereby achieving uniform compression of the fragment into the defect bed without penetrating the fragment itself [19, 31].

The purpose of this study was to evaluate, in patients with an acute patellar dislocation and a concomitant retropatellar osteochondral flake fracture, whether osteochondral flake refixation using the parachute technique combined with concomitant medial patellofemoral ligament (MPFL) reconstruction results in favourable clinical and radiological outcomes at a minimum follow‐up of 24 months. It was hypothesized that the technique would yield favourable clinical and functional outcomes and good osteochondral integration on magnetic resonance imaging (MRI).

## MATERIALS AND METHODS

Following institutional review board (IRB) approval of the Technical University of Munich (IRB 196/17S), a retrospective chart review was conducted to identify all patients who presented with an osteochondral flake fixation following an acute patellar dislocation between January 2012 and November 2023 at the authors' institution. Patients were eligible for inclusion if they had sustained a primary, acute patellar dislocation with a confirmed osteochondral flake fracture of the retropatellar cartilage surface and had been treated with the parachute technique in combination with concomitant soft tissue patellar stabilization. The indication for surgery and for any concomitant bony procedure was based on a standardized assessment of patellofemoral instability risk factors, comprising trochlear dysplasia (graded according to the Dejour classification), patellar height (Caton–Deschamps index), tibial tuberosity–trochlear groove (TT–TG) distance or tibial tuberosity posterior cruciate ligament insertion distance and patellar tilt. Refixation of the osteochondral fragment was indicated in all patients with an intact, displaced fragment and a defect of at least 1 cm^2^. Osteochondral fragments that were smaller than 1 cm^2^ or no longer amenable to refixation because of comminution or insufficient fragment quality were not treated with the parachute technique; depending on the intraoperative findings, these knees underwent fragment removal without refixation, an alternative cartilage‐restoring procedure or screw fixation, and the corresponding patients were therefore not part of the present cohort. All patients received concomitant MPFL stabilization to address the soft‐tissue insufficiency resulting from the acute dislocation. Open trochleoplasty according to Bereiter was reserved for patients with high‐grade trochlear dysplasia (Dejour Type C–D). Patients were excluded if they had a history of prior knee surgery at the index knee or had sustained a concurrent patellar fracture. A minimum follow‐up of 24 months was required, as maturation of the cartilage repair tissue and osseous integration of the fragment are generally considered to be sufficiently advanced by this time point to allow a meaningful MRI‐based assessment. All procedures were performed at a single academic institution by five experienced, fellowship‐trained knee surgeons and the surgical technique, the standardized rehabilitation protocol, and the perioperative pain‐management regimen remained unchanged throughout the study period. All patients provided written informed consent prior to participation. A detailed description of the surgical technique together with preliminary clinical results of this cohort has been previously published in German [31]; the present study reports the complete short‐ to mid‐term patient‐reported outcomes together with the standardized MRI‐based assessment of osteochondral integration (Magnetic Resonance Observation of Cartilage Repair Tissue [MOCART] 2.0), which were not part of that earlier report.

### Surgical technique

All patients underwent diagnostic arthroscopy to retrieve the osteochondral fragment (if arthroscopically possible), assess the defect bed and confirm the indication for refixation. Eligibility for refixation was defined as an intact sheared‐off fragment with adequate surrounding cartilage enabling secure embedding in the defect bed. Fragments that did not conform precisely to the defect bed were trimmed using a scalpel.

The mini‐open parachute technique was performed as follows: A central, paramedian skin incision was made, and dissection was carried down to the joint capsule at its transition to the vastus medialis. The joint capsule was incised using electrocautery to expose the retropatellar cartilage surface [19].

**Figure 1:**
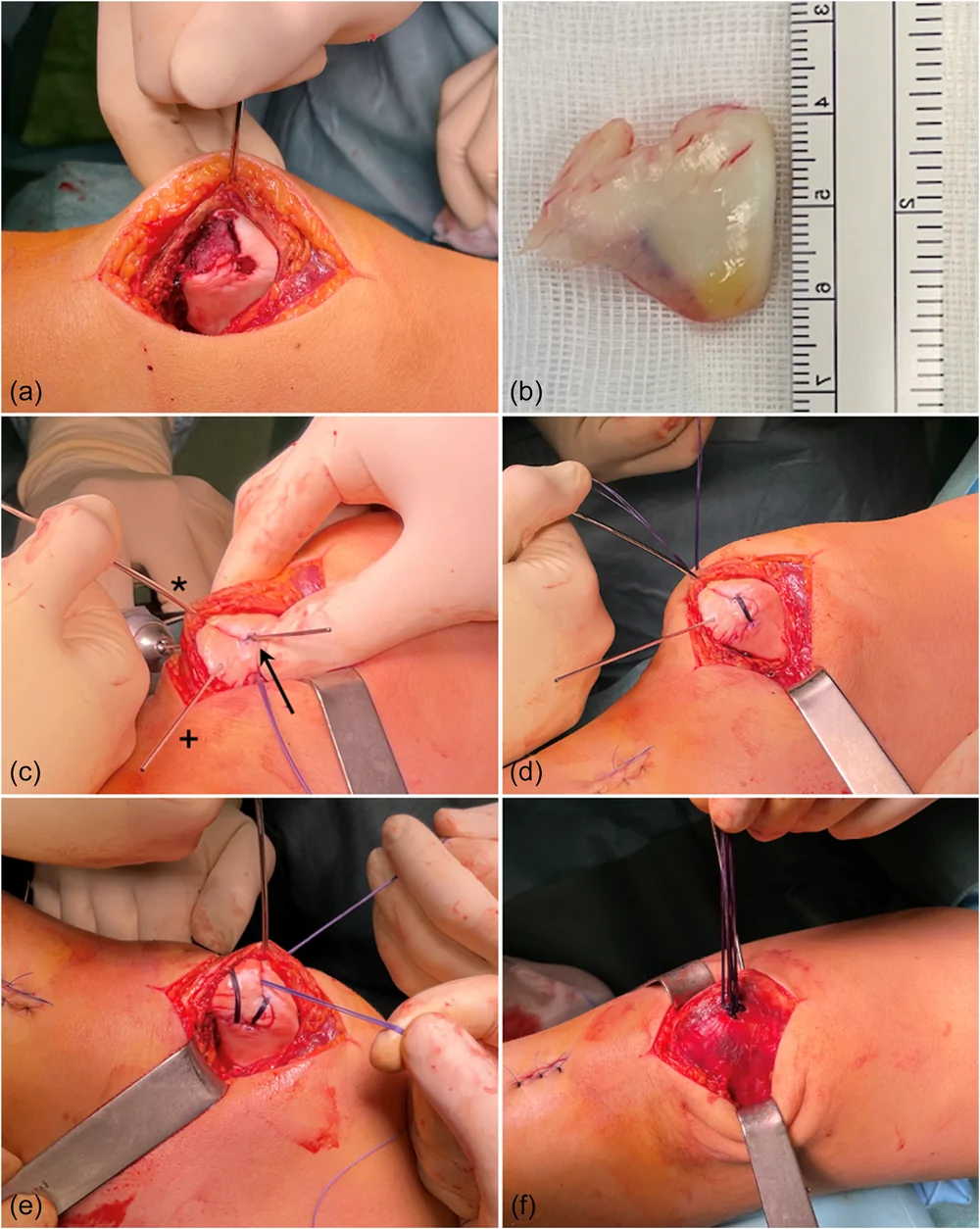
Parachute technique for osteochondral flake refixation following acute patellar dislocation of the left knee, performed via a mini‐open approach on the medial aspect of the patella. (a) The chondral defect is located at the inferomedial aspect of the patella. (b) In this case, the osteochondral fragment was retrieved from the joint and measured 25 × 25 mm. (c) A 2.0 mm Kirschner wire (K‐Wire) was drilled in the patella in a horizontal (*) fashion to create a lever to stabilize the patella during the procedure. A second 1.5 mm K‐wire was temporarily passed through the fragment (+) to stabilize it for subsequent steps, and a third K‐wire (2.0 mm) was placed through the patella at the junction of the defect bed and fragment margin (black arrow). (d) The K‐wire was advanced, shuttling Vicryl sutures (USP 1/0; Ethicon Inc.) through transpatellar tunnels that were directed to converge at the anterior patellar surface. (e) This step was repeated until a stable construct was achieved. (f) The sutures were tied on the anterior patellar surface using the technique described by Mehl et al. [19].

The defect bed was carefully debrided of haematoma using a sharp curette and unstable margins were resected. Transpatellar bone tunnels were created at the margins of the osteochondral fragment using a 1.4 mm Kirschner wire. Absorbable sutures (Vicryl USP 1/0, Ethicon Inc.) were passed through the transpatellar tunnels using a flexible nitinol suture passing wire (Arthrex). The tunnel entry points were positioned to converge at the anterior patellar cortex, creating a confluent configuration [19]. Four to six tunnels were drilled, depending on the size and shape of the flake, to achieve a stable suture configuration (Figure 1). All patients also underwent concomitant reconstruction of the MPFL using an autologous gracilis tendon during the same standard surgical procedure as previously described [22].

### Postoperative rehabilitation

All patients followed a standardized rehabilitation protocol. Partial weight‐bearing was limited to 20 kg for 6 weeks postoperatively. Range of motion (ROM) of the knee was progressively increased according to the following protocol: 0°/0°/30° during Weeks 1–2, 0°/0°/60° during Weeks 3–4 and 0°/0°/90° during Weeks 5–6. Full weight‐bearing and unrestricted ROM were permitted thereafter, with a gradual return to sport.

### Clinical outcome assessment

Clinical outcomes were assessed at final follow‐up using previously validated patient‐reported outcome measures (PROMs). The Visual Analogue Scale (VAS) was used to evaluate pain intensity. The Tegner Activity Scale (TAS) was used to assess the level of sporting and occupational activity [28]. The Kujala Score was used to measure anterior knee pain and patellofemoral function [5]. The Knee Injury and Osteoarthritis Outcome Score (KOOS) and its five subscores (Symptoms, Pain, Activities of Daily Living, Sport and Recreation and Quality of Life) were used to evaluate knee‐related impairment [25]. The International Knee Documentation Committee (IKDC) subjective score was used to assess overall knee symptoms, function and sporting activity [13]. Additionally, patients' overall satisfaction with the surgical result (yes/no), willingness to undergo the procedure again if they had the same injury (yes/no), and the recurrence of postoperative patellar dislocation (yes/no) were recorded.

### Radiological outcome assessment

**Figure 2:**
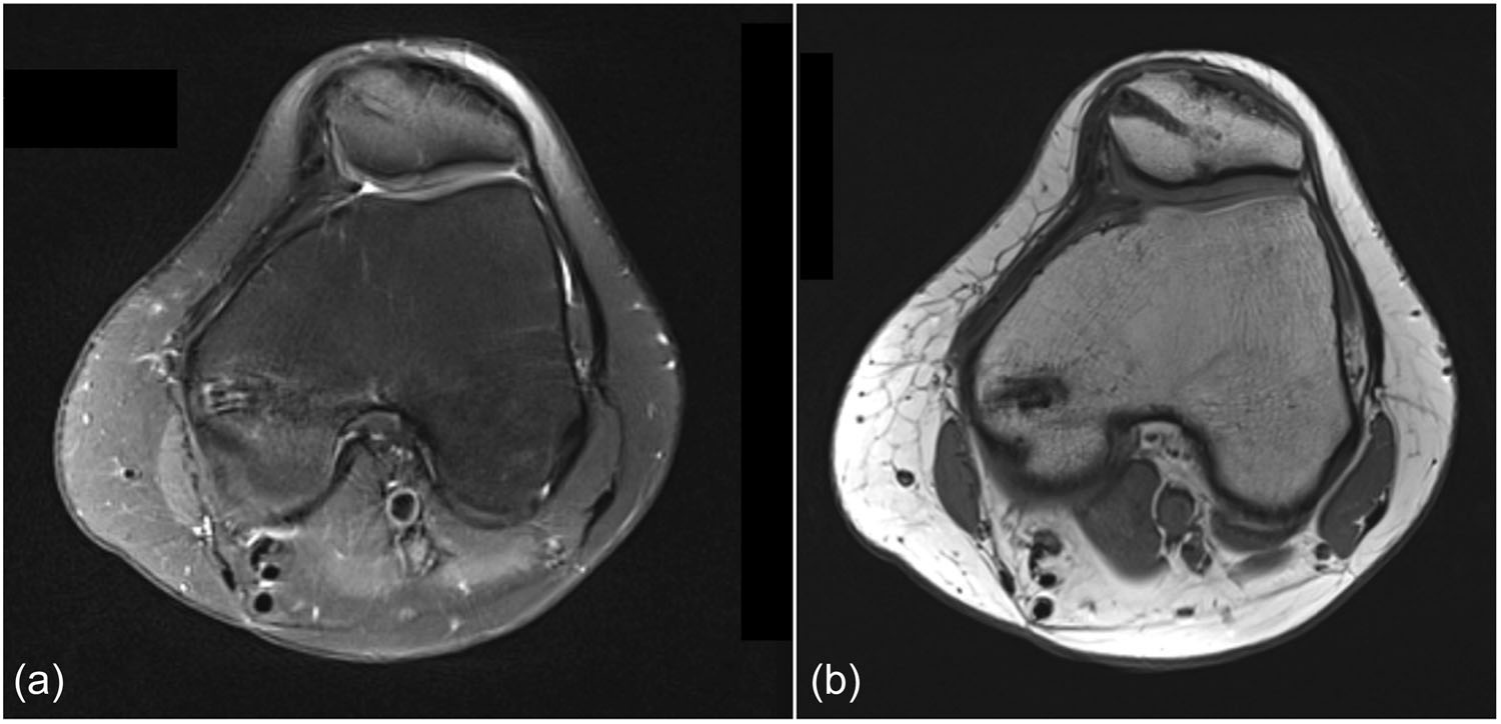
Postoperative magnetic resonance imaging (MRI) 2 years after performing the parachute technique in a right knee after traumatic patellar dislocation with an osteochondral flake fracture. Osteochondral integration is visible on the (a) axial T2‐weighted and (b) intermediate‐weighted turbo spin‐echo (TSE) images.

MRI of the affected knee was performed at final follow‐up. All MRI examinations were performed on a 3.0‐T MR scanner using a dedicated knee coil. Sagittal and axial intermediate‐weighted turbo spin‐echo (TSE) sequences with spectral fat saturation were obtained to evaluate the articular cartilage (Figure 2). Cartilage repair tissue was assessed semiquantitatively using the MOCART 2.0 knee score [27]. MR images were independently evaluated by three raters: one radiologist with dedicated musculoskeletal imaging experience (A. W. M.) and two orthopaedic surgeons (L. F. and R. P. V.). All three raters assessed the images independently and were blinded to the clinical outcomes and to one another's ratings. Inter‐rater reliability among all three raters was assessed using the intraclass correlation coefficient (ICC).

### Statistical analysis

Statistical analysis was performed using IBM SPSS Statistics (Version 30.0; IBM Corp.). Normal distribution of continuous variables was assessed using the Shapiro–Wilk test. Normally distributed data are presented as mean ± standard deviation (SD) with range. Categorical variables are reported as absolute counts and relative frequencies. Inter‐rater reliability for the MOCART 2.0 score was evaluated using the ICC: 0–0.2 represents slight agreement; 0.21–0.4 indicates fair agreement; 0.41–0.6 signifies moderate agreement; 0.61–0.8 shows substantial agreement and 0.81–1.0 indicates almost perfect agreement [1]. The level of significance was set at *α* = 0.05. As this was a descriptive case series of a rare injury, no a priori sample‐size calculation was performed; instead, all consecutive eligible patients treated during the study period were included. The sample size therefore reflects the available population rather than a predetermined target, and all analyses should be regarded as descriptive and hypothesis‐generating.

## RESULTS

**Figure 3:**
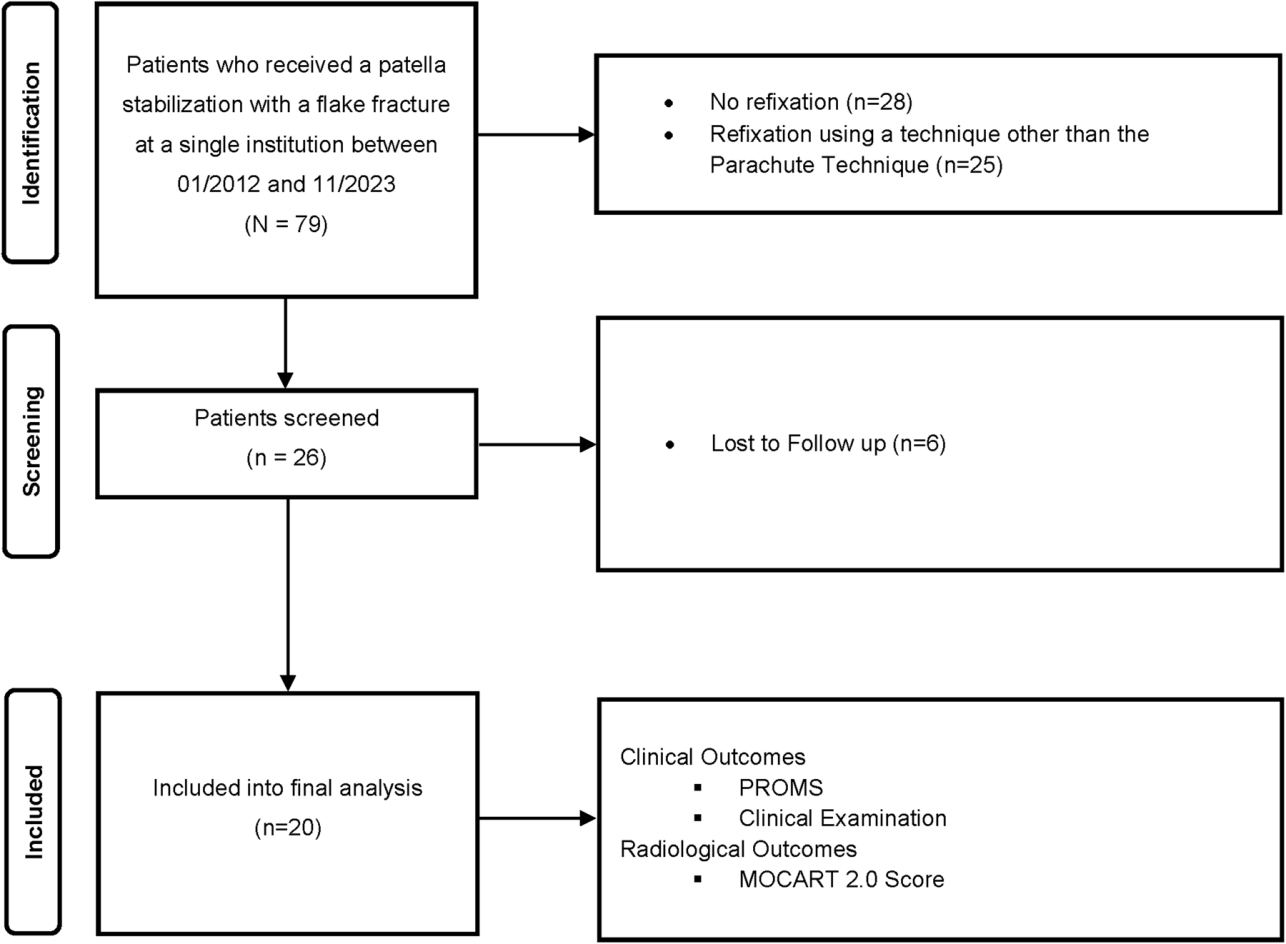
Flowchart of patient selection for clinical and radiological follow‐up examination. Patients who received an osteochondral flake refixation using the parachute technique were screened for eligibility. MOCART, Magnetic Resonance Observation of Cartilage Repair Tissue; *n*, number of subjects; *N*, total number of subjects; PROMs, patient‐reported outcome measures.

A total of 26 patients who underwent osteochondral flake refixation using the parachute technique following acute patellar dislocation between January 2012 and November 2023 were identified (Figure 3). Of these, 20 patients (77% follow‐up rate) were available for final follow‐up and constituted the study cohort. The mean follow‐up was 62.2 ± 20.5 months (range: 27.0–111.3). Mean defect size was 2.8 ± 1.5 cm^2^. Of 20 patients, 18 (90%) underwent MPFL reconstruction with an autologous gracilis tendon graft, and 1 (5%) underwent trochleoplasty during the same surgery. All concomitant procedures were patellar‐stabilizing procedures performed during the same single‐stage operation to address the patellofemoral instability resulting from the acute dislocation; they are reported separately (Table 1). Detailed demographic data are presented in Table 1.

**Table 1:** Preoperative demographic data, follow‐up, size of defect and concomitant procedures of the study cohort.

Parameter	Value	SD	Range
Age at surgery (years)	20.8	±5.3	14.0–30.6
BMI (kg/m^2^)	24.7	±6.7	18.1–51.3
Follow‐up (months)	62.2	±20.5	27.0–111.3
Sex (male/female)	10/10	—	—
Laterality (right/left)	11/9	—	—
Size of defect (cm^2^)	2.8	±1.5	1–6
Concomitant procedures			
MPFL reconstruction with autologous gracilis tendon graft (*n*)	18		
MPFL refixation (*n*)	2		
Trochleoplasty (open)	1		

*Note*: Continuous variables are presented as mean ± SD with range. One patient underwent both trochleoplasty and MPFL reconstruction; therefore, the sum of concomitant procedures exceeds the cohort size (*n* = 20).

Abbreviations: BMI, body mass index; cm^2^, square centimetre; kg, kilograms; MPFL, medial patellofemoral ligament; SD, standard deviation.

### Clinical outcome

All 20 patients completed the PROMs at final follow‐up. The mean VAS pain score was 0.5 ± 1.6 (range: 0–7), indicating minimal residual pain in the cohort. The mean Kujala Score was 89.4 ± 12.5 (range: 60–100) and the mean IKDC score was 86.7 ± 14.3 (range: 54–100). The mean total KOOS was 87.8 ± 14.1 (range: 52–100). The TAS was 6 (interquartile range 4–7), reflecting a return to moderate‐to‐high levels of physical activity. All clinical outcome scores are detailed in Table 2.

**Table 2:** Clinical outcome scores at final follow‐up.

Parameter	Mean/Median	±SD/IQR	Range
VAS	0.5	±1.6	0–7
TAS	6	4–7	2–9
Kujala Score	89.4	±12.5	60–100
IKDC	86.7	±14.3	54–100
KOOS Total	87.8	±14.1	52–100
KOOS Symptoms	84.6	±13.7	60–100
KOOS Pain	92.8	±11.5	58–100
KOOS ADL	94.6	±13.8	43–100
KOOS Sport & Recreation	84.7	±21.0	35–100
KOOS Quality of Life	81.9	±24.7	25–100

Abbreviations: ADL, Activities of Daily Living; IKDC, International Knee Documentation Committee; IQR, interquartile range; KOOS, Knee Injury and Osteoarthritis Outcome Score; SD, standard deviation; TAS, Tegner Activity Scale; VAS, Visual Analogue Scale.

Regarding patient satisfaction, 19 patients (95.0%) were satisfied with the overall clinical outcome at the time of follow‐up. A total of 18 patients (90.0%) stated they would undergo the procedure again if they had the same injury again. Two patients (10%) had to undergo revision surgery: One patient (male, 28 years) reported a traumatic redislocation of the patella, which was then treated with an isolated MPFL‐revision reconstruction with allograft. One patient (male, 23 years of age) required revision surgery 3 months after the index procedure due to failure of fragment integration and was subsequently treated with a cartilage regenerative procedure (Minced Cartilage). As only a single failure of fragment integration occurred, no reliable patient‐specific risk factor at the index procedure—such as defect size, lesion location, fragment thickness or the interval from injury to surgery—could be identified for this complication in the present cohort. Patient satisfaction and complication data are summarized in Table 3.

**Table 3:** Patient satisfaction, recurrence of patellar dislocation and revision rate at final follow‐up.

Item	Yes	No
Would you undergo the same procedure again if you had the same injury?	18 (90%)	2 (10%)
Satisfied with clinical outcome?	19 (95%)	1 (5%)
Recurrence of patellar dislocation?	1 (5%)	19 (95%)
Revision surgery required?	2 (10%)	18 (90%)

### Radiological outcome

MR imaging of the affected knee was performed in all 20 patients at final follow‐up. The mean MOCART 2.0 score was 84 ± 8 (range: 75–95), indicating good to excellent osteochondral integration of the fragment. Inter‐rater reliability among the three raters was almost perfect, with an ICC of 0.84 (95% confidence interval [CI] 0.79–0.96), the lower bound of the CI bordering the substantial range.

## DISCUSSION

The most important finding of this study was that the parachute technique yielded favourable clinical and radiological outcomes at short‐ to mid‐term follow‐up in patients with osteochondral flake fractures after acute patellar dislocation. The results of this study indicate good to excellent osteochondral integration of the fragment and overall high patient satisfaction.

Osteochondral fractures after acute patellar dislocation are frequently accompanied by joint effusion and limited ROM due to the dislocated fragment and remain a challenging entity in orthopaedic practice. There is consensus that large chondral and osteochondral lesions with a size of 1 cm^2^ should be repaired to retrieve the loose body and restore the articular surface [3]. The parachute technique is a single‐step procedure that uses absorbable sutures placed at the border of the fragment bed and the fragment itself. This technique aims to uniformly press the fragment into the defect bed without penetrating the fragment itself [19, 31]. The combination of the single‐stage procedure and preserving the fragments' integrity represents advantages compared to refixation techniques using screws or pins [7, 9].

For surgical therapy, various techniques have been described to restore osteochondral integrity [7, 9, 14, 15, 19, 20, 21]. Screw fixation provides rigid stabilization but requires penetration of both the fragment and the healthy cartilage, and metallic hardware necessitates a second surgical procedure for implant removal [7, 9, 14]. Bioabsorbable pins avoid the need for a second surgery but are associated with considerable implant costs and the risk of implant‐related complications, including foreign body reactions and pin breakage [7, 14]. Autologous chondrocyte implantation represents another established alternative for cases in which refixation is not feasible due to a small or unstable fragment [7]. However, autologous chondrocyte implantation may require a two‐stage procedure, and the available evidence on the stability and durability of the transplant in the context of large osteochondral defects of the patella after acute patella dislocation remains limited [10]. The parachute technique addresses several of these limitations by offering a simple, single‐stage surgical option that achieves stable fixation of the native osteochondral fragment using absorbable sutures, without the need to penetrate the fragment itself [19, 31]. This allows the original hyaline cartilage to be preserved and used to fill the defect, while the distributed suture configuration provides uniform compression across the fragment surface.

Although clinical outcomes following osteochondral flake refixation are generally reported to be satisfactory, radiological data evaluating the quality of cartilage repair tissue using validated MRI‐based assessment tools remain scarce [7, 14, 30]. The current study addresses this gap by providing semiquantitative MRI evaluation of osteochondral integration using the MOCART 2.0 score in a series of 20 patients treated with a suture‐based refixation technique for large osteochondral fragments. Whereas the surgical technique and preliminary results of this cohort were reported previously in a German‐language technique article [31], the present study extends that work by adding the complete short‐ to mid‐term patient‐reported outcomes and, for the first time, a standardized MRI‐based evaluation of osteochondral integration, thereby complementing rather than duplicating the earlier report. The MOCART 2.0 scores observed in the present cohort reflect good to excellent integration independent of the fragment size and are in line with previously published radiological results following open refixation [7]. When fragment size was explored against radiological and clinical outcomes, no clear association with MOCART 2.0 scores or with the need for revision was observed; given the small cohort, this observation should be regarded as exploratory and hypothesis‐generating. One patient required revision surgery due to failure of fragment integration and subsequently underwent autologous chondrocyte implantation, consistent with the recommended treatment algorithm for failed refixation [3, 19].

Clinical outcomes following osteochondral flake refixation after acute patella dislocation are reported to be good, with high rates of return to previous activity levels [9, 14]. Because 90% of the cohort underwent concomitant MPFL reconstruction, with a further two MPFL refixations and one trochleoplasty, the favourable clinical scores cannot be attributed to the osteochondral refixation technique in isolation; they should rather be interpreted as reflecting the combined effect of cartilage restoration and concomitant patellofemoral stabilization. Concomitant patellofemoral stabilization has been studied extensively: Comparable clinical outcomes have been reported for hamstring and quadriceps tendon autografts in MPFL reconstruction [18], combined trochleoplasty and MPFL reconstruction have been shown to reduce patellar height [24], and trochleoplasty remains among the most extensively studied bony stabilizing procedures for patellofemoral instability. The favourable results observed in the present study and in the existing literature may in part be attributed to the characteristics of the patient population: Affected individuals are typically young and otherwise healthy, with intact surrounding cartilage and good biological healing potential. Furthermore, the presence of subchondral bone on the fragment facilitates vascular ingrowth and osseous integration, which may explain why even larger fragments tend to heal reliably when adequately stabilized [14]. In contrast to chronic patellofemoral instability, where anatomical risk factors typically play an important role, osteochondral fractures are usually caused by a traumatic injury mechanism [6]. The findings of the present study support this notion, as after individual risk assessment of each patient, only one patient underwent concomitant trochleoplasty according to Bereiter due to high‐grade trochlear dysplasia [32].

The present study has several limitations that should be considered. First, the MOCART 2.0 score was used as a surrogate parameter for osteochondral integration, and while it provides a validated and standardized semiquantitative assessment of cartilage repair tissue, it does not allow direct histological evaluation of tissue quality. Second, this study represents a relatively small case series, which reflects the rarity of this injury and the strict inclusion criteria applied; a larger cohort would provide greater statistical power and allow for subgroup analyses. Third, the retrospective design does not include preoperative PROM data, precluding a formal assessment of the magnitude of clinical improvement. However, as all patients reported a traumatic first dislocation event in a previously healthy knee, it is reasonable to assume that baseline function prior to injury was unimpaired. Therefore, postoperative patient‐reported outcomes remain a meaningful and valid indicator of treatment success, as they can be interpreted against an assumed pre‐injury baseline of full functional capacity. Fourth, the mid‐term follow‐up does not allow conclusions regarding the long‐term risk of patellofemoral osteoarthritis, which remains one of the primary concerns following cartilage injury in this patient population [2]. Fifth, 6 of the 26 eligible patients (23%) were lost to follow‐up, which may have introduced selection bias; a comparison of baseline characteristics between patients who were and were not available for follow‐up was not feasible, and the possibility that outcomes differed between these groups cannot be excluded. Sixth, the procedures were performed by five different surgeons over the study period; although the surgical technique and the standardized rehabilitation and pain‐management protocols remained unchanged throughout, a potential influence of inter‐surgeon variability on the outcomes cannot be entirely excluded. Finally, the absence of a comparator group limits the strength of the conclusions that can be drawn and precludes any direct comparison with alternative fixation techniques. Future prospective studies with longer follow‐up periods are warranted to address these limitations.

## CONCLUSION

The parachute technique yields favourable clinical and functional outcomes at short‐ to mid‐term follow‐up and demonstrates good to excellent osteochondral integration on MRI following acute patellar dislocation. The technique represents a reliable, simple and single‐stage option for the treatment of retropatellar osteochondral flake fractures.

## AUTHOR CONTRIBUTIONS

*Conceptualization*: Romed Peter Vieider and Julian Mehl. *Methodology*: Romed Peter Vieider, Yannick Ehmann and Julian Mehl. *Data acquisition*: Romed Peter Vieider, Yannick Ehmann, Svenja Höger, Alexander Wolfgang Marka, Lorenz Fritsch and Armin Runer. *Writing—original draft preparation*: Romed Peter Vieider. *Writing—review and editing*: All authors. *Supervision*: Sebastian Siebenlist and Julian Mehl.

## FUNDING INFORMATION

The authors have no funding to report.

## CONFLICT OF INTEREST STATEMENT

Julian Mehl declares consulting fees from Arthrex GmbH and Ormed GmbH. Sebastian Siebenlist declares consulting fees from Arthrex GmbH, KLS Martin Group and Medi GmbH & Co. KG. The remaining authors declare no conflict of interest.

## ETHICS STATEMENT

Ethical approval was obtained from the Institutional Review Board of the Technical University of Munich (IRB 196/17S). Written informed consent was obtained from all patients prior to participation.

## Supporting information


**Supplementary Material.** STROBE Checklist.

## Data Availability

The data that support the findings of this study are available on request from the corresponding author. The data are not publicly available due to privacy or ethical restrictions.
