# Supplementary material for: Osteochondral flake refixation using the parachute technique yields favourable clinical and radiological outcomes following acute patellar dislocation: A case series at short‐ to mid‐term follow‐up
Source: J Exp Orthop. 2026 Sep 9;13(3):e70894. doi: 10.1002/jeo2.70894 (PMC13556305; doi:10.1002/jeo2.70894)
Supplement: Supplementary file 1 [file JEO2-13-e70894-s001.docx]

# STROBE Statement — Checklist of items for reports of observational studies

**Study design:** Retrospective case series (Level of Evidence IV)

**Manuscript:** Osteochondral Flake Refixation Using the Parachute Technique Yields Favorable Clinical and Radiological Outcomes Following Acute Patellar Dislocation: A Case Series at Short- to Mid-Term Follow-up

| No. | Recommendation | Reported in manuscript |
| --- | --- | --- |
| **Title and abstract** |  |  |
| 1 | (a) Study design indicated in the title or abstract; (b) informative and balanced summary in the abstract | Title states “A Case Series”; structured Abstract (Purpose/Methods/Results/Conclusion) |
| **Introduction** |  |  |
| 2 | *Background/rationale:* scientific background and rationale | Introduction |
| 3 | *Objectives:* specific objectives and any pre-specified hypotheses | Introduction (aim and hypothesis, structured along PICOT) |
| **Methods** |  |  |
| 4 | *Study design:* present key elements early in the paper | Methods (retrospective case series) |
| 5 | *Setting:* setting, locations, and relevant dates (recruitment, exposure, follow-up, data collection) | Methods (single academic institution; January 2012 – November 2023) |
| 6 | *Participants:* eligibility criteria, sources, and methods of selection | Methods (inclusion/exclusion criteria; technique-selection by fragment suitability; Figure 3 flowchart) |
| 7 | *Variables:* outcomes, exposures, predictors, confounders, effect modifiers | Methods (clinical and radiological outcome assessment) |
| 8 | *Data sources/measurement:* sources and methods of assessment for each variable | Methods (validated PROMs; MOCART 2.0 on 3.0-T MRI) |
| 9 | *Bias:* efforts to address potential sources of bias | Methods and Limitations (blinded, independent MRI raters; loss to follow-up and selection bias addressed) |
| 10 | *Study size:* how the study size was arrived at | Methods (all consecutive eligible patients; no a priori sample-size calculation, rationale given) |
| 11 | *Quantitative variables:* handling of quantitative variables and groupings | Methods (Statistical analysis) |
| 12 | *Statistical methods:* all methods, subgroup/interaction, missing data, loss to follow-up, sensitivity analyses | Methods (Statistical analysis; ICC for inter-rater reliability) |
| **Results** |  |  |
| 13 | *Participants:* numbers at each stage, reasons for non-participation, flow diagram | Results and Figure 3 (26 identified, 20 available at follow-up; 6 lost to follow-up) |
| 14 | *Descriptive data:* characteristics of participants; missing data; follow-up time | Results and Table 1 (demographics, defect size, follow-up 62.2 ± 20.5 months) |
| 15 | *Outcome data:* numbers of outcome events or summary measures | Results and Tables 2–3 |
| 16 | *Main results:* estimates and precision (e.g., 95% CI) | Results (means ± SD, ranges; ICC with 95% CI) |
| 17 | *Other analyses:* subgroups, interactions, sensitivity analyses | Discussion (exploratory analysis of fragment size vs. outcomes) |
| **Discussion** |  |  |
| 18 | *Key results:* summary with reference to study objectives | Discussion (opening paragraph) |
| 19 | *Limitations:* limitations, sources of bias/imprecision, direction and magnitude | Limitations (design, sample size, absence of comparator, loss to follow-up, inter-surgeon variability) |
| 20 | *Interpretation:* cautious overall interpretation considering objectives, limitations, and other evidence | Discussion |
| 21 | *Generalisability:* external validity | Discussion and Limitations (selected subgroup of intact, refixable fragments) |
| **Other information** |  |  |
| 22 | *Funding:* sources of funding and role of funders | Title page (Funding statement; conflict-of-interest disclosure) |

*Adapted from: von Elm E, Altman DG, Egger M, Pocock SJ, Gøtzsche PC, Vandenbroucke JP. The Strengthening the Reporting of Observational Studies in Epidemiology (STROBE) statement: guidelines for reporting observational studies. J Clin Epidemiol. 2008;61(4):344–349.*
